# Experimental investigations of social exclusion among adolescents with psychiatric disorders: a systematic review

**DOI:** 10.1007/s00787-025-02687-9

**Published:** 2025-04-30

**Authors:** Lior Weinreich, Kristina Moll, Matthias F. J. Sperl, Gerd Schulte-Körne, Bert Timmermans

**Affiliations:** 1 Department of Child and Adolescent Psychiatry, Psychosomatics and Psychotherapy, University Hospital, Ludwig Maximilian University of Munich, Nussbaumstrasse 5, Munich, 80336 Germany; 2 Department of Clinical Psychology and Psychotherapy, University of Siegen, Siegen, Germany; 3 Department of Clinical Psychology and Psychotherapy, University of Giessen, Giessen, Germany; 4 School of Psychology, University of Aberdeen, Aberdeen, Scotland

**Keywords:** Social exclusion, Adolescents, Psychiatric disorders, Mental disorders

## Abstract

Social exclusion is a form of bullying that can lead to various negative consequences, and even extreme forms of violence. Certain groups, such as people with poor mental health and adolescents, are particularly vulnerable. This paper features a systematic review of experiments that investigated the impact of social exclusion on adolescents with psychiatric disorders. Experiments were searched via: PubMed, Web of Science, PsycInfo, ERIC, Cochrane, and a manual search. The search yielded 174 experiments, and 12 remained after screening. These met the inclusion criteria, which included: having an empirical design, participants aged 10–19, and a clinical sample with at least one psychiatric disorder. Among the clinical samples, the most common disorder was depression, featured in seven experiments. The most common paradigm was Cyberball. Results showed that social exclusion impacts adolescents with psychiatric disorders differently than inclusion (e.g., leading to a more negative mood). However, the difference in the impact of social exclusion on adolescents with vs. without psychiatric disorders was only conclusive via fMRI measurements. Compared to healthy controls, adolescents with psychiatric disorders seem to display altered neural reactivity during social exclusion. Based on identified research gaps, future studies are needed to explore the impact of social exclusion on adolescents with a wider range of psychiatric disorders. Other recommendations are included, such as a brain region checklist for future experiments using fMRI.

**Supplementary Information:**

The online version contains supplementary material available at 10.1007/s00787-025-02687-9.

## Introduction

Social exclusion is an umbrella term, which usually refers to bullying through exclusion from social relationships, occasionally accompanied by statements of dislike [1]. Rejection, on the other hand, is a term that refers to exclusion from a group by either being teased, ignored, or experiencing unrequited love [2]. Ostracism, conversely, is a term that refers to exclusion done without any explanation or indication of negative intentions (i.e., being ignored with no apparent reason [3]. Despite these distinctions, in the vast body of research investigating the impact of social exclusion, these terms are often used interchangeably [3]. The current systematic review will use the term social exclusion predominantly, to denote exclusion from a social group of peers.

But why is social exclusion vastly researched? In other words, why is it important? According to the evolutionary perspective, social exclusion plays a vital role in social relationships that, in turn, impact survival [3]. This claim is detailed in the theoretical work by MacDonald and Leary [4]. Their work centers around the notion that social exclusion causes a condition of discomfort. This discomfort shares certain neural response patterns, such as greater activation in the anterior cingulate cortex, with pain caused by physical injuries [5]. Thus, it is often referred to as “social pain” ([6]; for a critical perspective on this see [7, 8]). When social animals experience the so-called “social pain”, it prompts them to react against threats to inclusion. For humans, inclusion in supportive social relationships promotes survival [9]. Moreover, MacDonald and Leary [4] give examples from monkey studies, which demonstrate that monkeys that form strong social relationships are more likely to survive and reproduce (e.g., [10]). The authors stress that similarly to monkeys, identifying social exclusion and reacting to it was key to our ancestors’ survival.

Furthermore, being socially excluded can lead to various negative consequences, such as threats to one’s self-esteem [11], increased risk at developing both internalizing and externalizing problems [12], reduction in prosocial behavior, and an overall induction of a negative emotional state [13–15]. Moreover, social exclusion can leave adolescents with the sense that they have been unjustly humiliated, leading to feelings of embitterment [16]. One alarming possible consequence is violent behavior [16]. In fact, the association between social exclusion and violent behavior has been supported in experimental settings [1]. Notably, violent affinity tends to be at its peak during adolescence [17]. Leary et al. [2] demonstrated that chronic rejection, by either being socially excluded, bullied, or experiencing unrequited love, is a common denominator in adolescents that committed school shootings. Their findings suggest that social exclusion on its own is usually not a risk factor. However, combined with one or more of the risk factors for school shooting (i.e., interest in weapons, psychiatric disorders, fascination with “dark themes” like death and Satan) can lead to violent behavior towards peers. For example, in 1997, 14-year-old Michael Carneal, a teen with a history of psychiatric difficulties, shot and killed three peers and injured five others. After the shooting he reported feeling rejected and disrespected at school. Strikingly, the first person he shot was the object of his unrequited love [18]. Survivors of school shootings, on the other hand, often suffer from negative outcome such as academic difficulties and an increased risk of major depression [19, 20].

Some individuals, such as people with poor mental health, are more vulnerable to the negative consequences of social exclusion. For example, Seidl et al. [21] showed that adults with a borderline personality disorder (BPD) reported a lower sense of belonging, meaningful existence, self-esteem, and control after being ostracized compared to a control group. The authors explained this by the reinforcement of pre-existing interpersonal difficulties. Moreover, Reinhard et al. [22] proposed a “vicious cycle”, wherein having psychopathologies increases the likelihood of social exclusion, which in turn increases symptom manifestation.

Additionally, age seems to play a role in social exclusion vulnerability, as demonstrated by studies comparing adolescents to other age groups. For example, Sebastian et al. [23] compared healthy adolescents and adults (i.e., with no history of neurological or psychiatric disorders), and found that following social exclusion adolescents reported a greater negative mood. The authors explained this by the fact that the ability to regulate distress caused by social exclusion and its related neural functions are developing during life’s second decade. Furthermore, during adolescence, peer-perceived status plays a powerful role [24]. This corresponds with the developmental trajectory of preferred companionship [25]. According to this trajectory, throughout late childhood and adolescence, there is an incremental shift from the preferred companionship of family members to that of peers.

Adolescents with psychiatric disorders are particularly vulnerable to social exclusion, which even in mild cases, increases the likelihood of symptom manifestation (e.g., [26]). This could be explained by the “vicious cycle” Reinhard et al. [22] proposed. Specifically, ample research has shown that adolescents with psychiatric disorders (e.g., anxiety, depression, ADHD, etc.) are more likely to suffer from bullying victimization by their peers (notable examples include [11, 27–32]; for systematics reviews see [33, 34]). In turn, being bullied during adolescence increases the risk of psychiatric disorders (e.g., eating disorders and depression, for systematic reviews see [35–37]). Furthermore, a history of being bullied can increase negative affective responses and neural sensitivity to social exclusion [38–40]. Thus, further perpetuating the above-mentioned “vicious circle”.

Cyberball is the most commonly used paradigm for the experimental investigations of social exclusion among adolescents [41]. Cyberball was developed by Williams & Jarvis [42], and has been used in numerous experiments since its launch. Essentially, it is a ball tossing computer game. Participants are made to believe that they are playing with other players. The other players, in fact, are controlled by the experimenters. According to pre-decided conditions, the experimenters could induce social exclusion or inclusion, by preventing or allowing the ball to be passed to the participants. This seemingly simple paradigm has produced profound effects in multiple experimental investigations of social exclusion among adolescents (e.g., [43]). However, most of these experiments used a sample of typically developed adolescents [41]. Consequently, the effects of social exclusion on adolescents with psychiatric disorders are under-researched, although they are particularly vulnerable [34].

The objective of the current study is to systematically review experimental investigations of social exclusion among adolescents with psychiatric disorders. To the best of our knowledge, this has not been done before. The noteworthy systematic review by Beckman et al. [33], found an overall higher prevalence rate of cyberbullying in studies investigating children with neurodevelopmental disorders. Another noteworthy systematic review by Alhaboby et al. [44], found an overall higher risk of bullying victimization and psychiatric impact (i.e., mainly depression) in studies investigating adults with chronic conditions and disabilities. Moreover, the important systematic review by Reinhard et al. [22] contributed to the understanding of the manner in which adults with psychiatric disorders are impacted by social exclusion. Nevertheless, the above-mentioned reviews either did not distinguish social exclusion from other forms of victimization, or did not distinguish adolescents from other age groups.

In accordance with the PRISMA statement ([45]; see Appendix for a filled-out checklist), the Population, Intervention, Comparison, Outcomes, and Study (PICOS) parameters were defined for the included experiments. Specifically, the population was comprised of adolescents with psychiatric disorders, with a mean age between 10 and 19 years old, and with no geographical restriction. Moreover, the sample characteristics were detailed separately for the clinical and control groups in terms of type of disorder, age, and gender. The study intervention (i.e., experimental paradigm) was social exclusion. Additionally, the type of paradigm, the experimental design, and the conditions were detailed. The comparison was between conditions (i.e., social exclusion vs. inclusion / baseline). The outcomes were: (a) impact of social exclusion (i.e., social exclusion vs. inclusion / baseline), and / or (b) impact on clinical vs. control sample.

## Methods

### Protocol

The review follows a pre-defined protocol (see Appendix). Like the systematic review by Beckman et al. [33], the protocol begins with the study aim. It then continues with other items mentioned in the PRISMA checklist that should be reported in a systematic review [45]. These items are: the eligibility criteria, information sources, study selection, data collection process, data items, risk of bias in individual studies, and search strategy.

### Inclusion and exclusion criteria

The inclusion criteria for the experiments used in the final synthesis were: (a) empirical studies written or translated to English; (b) published as journal articles or dissertations; (c) the sample included a clinical population of adolescents aged 10–19 (i.e., the age range for adolescence defined by the World Health Organization [46]), with at least one psychiatric disorder; (d) social exclusion was experimentally induced; (e) the outcome data was on the impact of social exclusion vs. inclusion or a baseline condition.

The exclusion criteria for the experiments used in the final synthesis were: (a) studies that did not meet our inclusion criteria (e.g., non-empirical); (b) the sample mixed adolescents with other age groups, and the different age groups were not analyzed separately; (c) the outcome data was on the impact of witnessing social exclusion rather than experiencing it.

### Procedure

The procedure follows a pre-defined protocoled search strategy (see Appendix). Databases for health-care, behavioral, social, biomedical, educational, and life science were searched. Specifically, the used databases were: PubMed, Web of Science, PsycInfo, ERIC, and Cochrane. Additional records were also identified through Google Scholar and reference search. The search started on May 3rd 2023, and ended on November 11th 2023. There was no defined starting date for publication. All included experiments were published before the search ended. One exception was an experiment that was first included as a preprint, and published at a peer-reviewed journal at a later point [47].

## Results

### Study selection

**Fig. 1:**
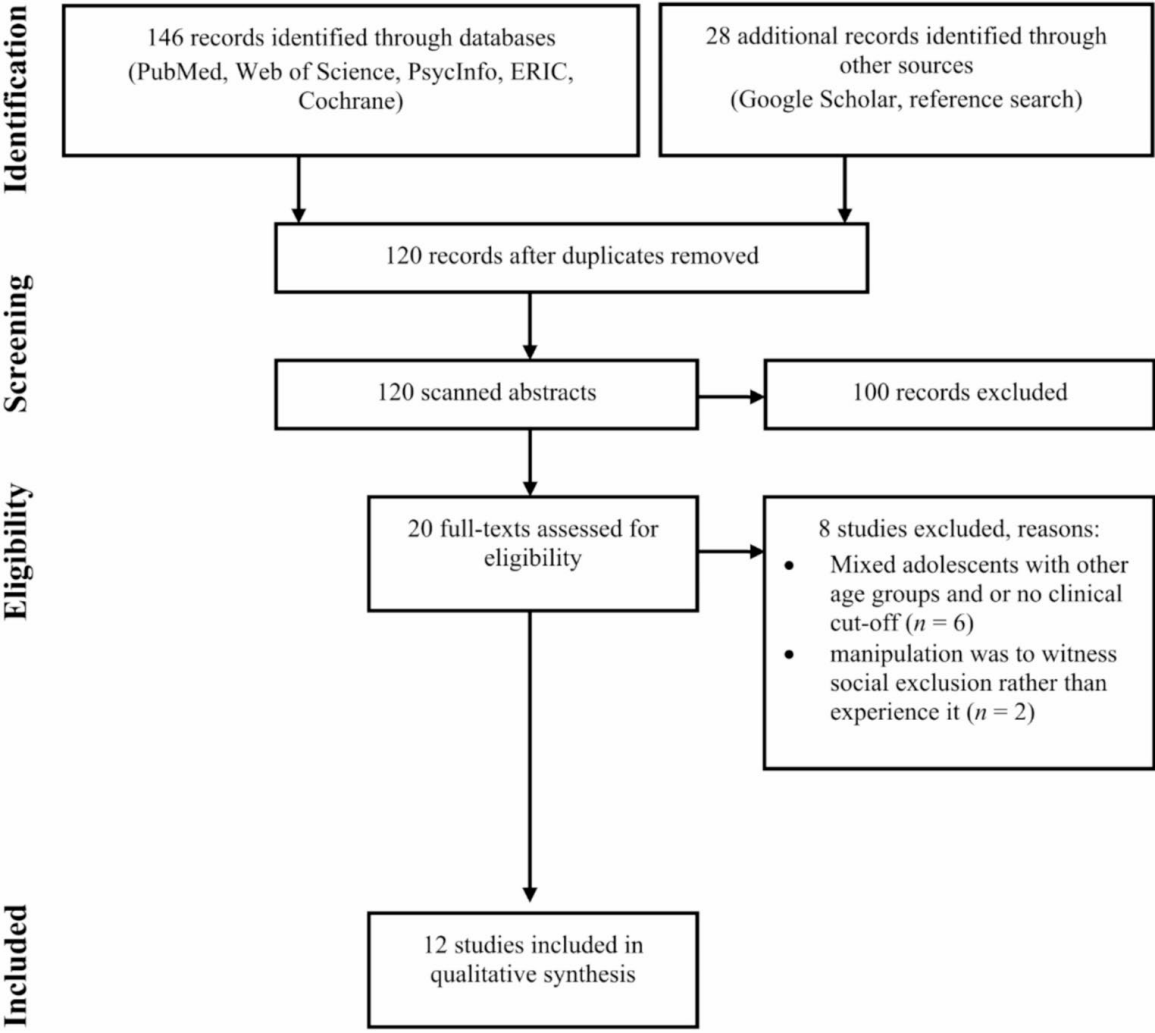
Flow diagram of the study selection process

The initial search yielded 174 records. After deduplication, 120 records remained. The abstracts of these records were read by the first author, leading to the exclusion of 100 records due to irrelevance to the current topic (e.g., adult studies). The full texts of the remaining 20 records were read by first and last authors. Each of these authors independently evaluated which of these records fulfilled the inclusion criteria. Both authors fully agreed on the 12 included records, as well as on the excluded records. Eight records were excluded for the following reasons: the sample mixed adolescents with other age groups, and the different age groups were not analyzed separately [48–52]; the outcome data was on the impact of witnessing social exclusion rather than experiencing it [53, 54]; the sample did not include a clinical population but rather the experimenters evaluated clinical traits [49, 55]. For an illustration of this process please see Fig. 1.

### Study characteristics

For a summary of the study characteristics please see Table 1.

#### Population

Taken together, all 12 included studies had a combined sample size of 758 participants. The smallest sample size was *N* = 26 [56], and the largest was *N* = 126 [57]. The youngest mean age (*M*age) among adolescent participants was 12 years old [58], and the oldest was 17 years old [59]. Among the 12 included studies, the most investigated psychiatric disorder was depression, with or without non-suicidal self-injury (NSSI), prior suicide attempt, and a comorbid borderline personality disorder (BPD). Specifically, depression was the common denominator in the clinical groups in seven studies [47, 57, 59–63]. Two other studies investigated clinical groups with autism spectrum condition (ASC; [56, 64]). The remaining three studies investigated clinical groups with other disorders. One study focused on attention deficit hyperactivity disorder (ADHD; [58]), and another focused on BPD [65]. Lastly, Latina et al. [66] focused on various psychiatric disorders and mental health conditions (i.e., NSSI, depression, social phobia, eating disorders, trauma, paranoid schizophrenia, emotional disorders, and multiple diagnoses), which they grouped under the umbrella term “emotional dysregulation”.

#### Intervention (Experimental Paradigm)

Among the 12 included studies, the most used paradigm was Cyberball [42], in combination with or without other paradigms. Specifically, Cyberball was used to induce social exclusion in 10 studies [47, 56–62, 64, 66]. The remaining two studies investigated social exclusion with script-driven imagery [65] and an interactive chat room task [63].

#### Comparison

All 12 included studies compared the impact of a social exclusion condition with that of an inclusion condition (e.g., in Cyberball by preventing or allowing a ball to be passed to participants). Four studies also included comparisons with a quasi-baseline condition (e.g., in Cyberball by having participants passively watch the game before playing; [56, 59, 60, 62]). Notably, Latina et al. [66] focused on the comparison between the impact of the commonly used paradigm Cyberball [42], to their newly developed task called Simulated On-Line Ostracism (SOLO).

#### Outcomes

Among the 12 included studies, the most used behavioral and neurophysiological outcome measurements were the Needs-Threat-Scale (NTS; [67]) and functional Magnetic Resonance Imaging (fMRI), respectively. The NTS was used in five studies [47, 56, 59, 60, 64], to assess the impact of social exclusion on participants’ self-reported sense of belonging, self-esteem, control, and meaningful existence. fMRI was used in eight studies [47, 57, 59–61, 63–65], as a measure of neural correlates associated with social exclusion. In the reviewed fMRI studies, within-subject contrast maps were typically derived using block-designed models (e.g., exclusion block > inclusion block, exclusion block > baseline block). Moreover, between-subjects differences in brain activation patterns (e.g., depression > HC) were further explored. Other notable outcome measurements included changes in heart rate and heart rate variability [66], as well as participants’ performance in an emotion recognition task [62]. Only four studies assessed identical measures before and after the inclusion / exclusion manipulation [56, 58, 62, 66].

#### Study

All 12 included studies employed a within-subject cross-sectional design. All studies apart form one [62] matched the clinical group with a healthy control group. Six studies were conducted in Germany, five in North America, and one in the UK.

**Table 1:** Study characteristics summary

Study, intervention, & comparison	Population	Outcomes	
		Impact of exclusion	Impact of exclusion on adolescent clinical vs. healthy control (HC) group
Brown et al. (2017) [59] Germany Cyberball Only watching Cyberball (baseline) vs. Inclusion via Cyberball vs. Exclusion via Cyberball	Adolescent depression + non-suicidal self-injury (NSSI): *n* = 13 *M*age = 15.5 10 females Adolescent control: *n* = 15 *M*age = 14.5 12 females Adult depression + NSSI + borderline personality disorder (BPD): *n* = 14 *M*age = 23.6 14 females Adult control: *n* = 17 *M*age = 23.2 17 females	fMRI comparing passive viewing, inclusion, and exclusion showed differential activation in the ventrolateral prefrontal cortex, pregenual anterior cingulate cortex, dorsal anterior cingulate cortex, ventral striatum, and the dorsolateral prefrontal cortex	The Hurt Feelings Scale (HFS) but not the Needs-Threat-Scale (NTS) results showed that adolescents with depression and NSSI self-reported more negative feelings compared to healthy controls (HC), indicating higher general sensitivity for social exclusion fMRI exclusion > inclusion contrasts showed that adolescents with depression + NSSI had higher activation of the putamen compared to both the depression + NSSI + BPD group and HC fMRI exclusion > baseline contrasts showed that adolescents with depression + NSSI had lower activation of the premotor cortex and dorsomedial prefrontal cortex compared to the depression + NSSI + BPD group; but higher activation of the putamen compared to HC, potentially indicating increased neural reactivity (salience network) and more intense negative social feedback processing related to NSSI, consistent with research conceptualizing bullying as an NSSI risk factor [90]
Gifuni et al. (2024) [47] Canada Cyberball + Go-NoGo to measure response inhibition Only watching Cyberball (baseline) vs. Inclusion via Cyberball vs. Exclusion via Cyberball 2 Go-NoGo block types: 3 “Go” blocks 3 “NoGo” blocks	Depression + prior suicide attempt: *n* = 29 *M*age = 16.3 25 females Depression + no prior suicide attempt: *n* = 35 *M*age = 16.0 28 females Control: *n* = 32 *M*age = 15.3 22 females	NTS results showed that adolescents with depression both with and without a prior suicide attempt had lower scores than HC on all subscales (Belonging, Self-Esteem, Significant Existence, Sense of Control)	
		fMRI exclusion > baseline contrasts showed that adolescents with depression + prior suicide attempt had: (1) lower activation in the right inferior frontal gyrus and higher activation in the right middle / superior frontal gyrus compared to adolescents with depression and no prior suicide attempt, and (2) lower activation in the left and right inferior frontal gyri compared to HC; also, (3) adolescents with depression and no prior suicide attempt had a lower activation in the left inferior frontal gyrus and the right middle / superior frontal gyrus compared to HC; consistent with research linking activation differences in these prefrontal brain areas to difficulties in emotion regulation [72] fMRI exclusion > inclusion contrasts showed that adolescents with depression and no prior suicide attempt had lower activation in the right precuneus and bilateral middle frontal gyrus compared to both other groups	
Groschwitz et al. (2016) [60] Germany Cyberball Only watching Cyberball (baseline) vs. Inclusion via Cyberball vs. Exclusion via Cyberball	Depression without NSSI: *n* = 14 *M*age = 15.9 11 females Depression + NSSI: *n* = 14 *M*age = 15.4 11 females Control: *n* = 15 *M*age = 14.5 12 females	The NTS indicated that participants felt excluded and distressed following social exclusion; fMRI exclusion > inclusion showed activation in the anterior insula, anterior cingulate cortex, parahippocampus, but also pre-supplementary motor area and secondary visual regions	The RSQ (Rejection Sensitivity Questionnaire) indicated elevated sensitivity to rejection in both depression groups; the NTS results showed no group differences apart from a greater feeling of helplessness among adolescents with depression + NSSI compared to HC; increased sensitivity to social rejection and similar feelings of social exclusion were evident in both depression groups, irrespectively of NSSI; results are consistent with the meta-analytical findings that bullying and social rejection seem to be critical risk factors for adolescent depression [87], contributing to a vicious circle of reciprocal influences that further stabilize the persistence of peer victimization and depressive symptoms [86] fMRI exclusion > inclusion contrasts showed that adolescents with depression + NSSI had greater activity in the medial prefrontal cortex, ventrolateral prefrontal cortex, and parahippocampus, compared to depression without NSSI; for peak voxel activation, differential medial prefrontal cortex / ventrolateral prefrontal cortex activity for depression + NSSI was higher than for both other groups, pointing toward altered processes in brain regions that have been associated with emotion regulation [72]
Hartmann et al. (2013) [58] Germany Stop signal task (SST) + Cyberball SST vs. Exclusion via Cyberball vs. SST	Loss of control (LOC) eating: *n* = 23 *M*age = 12 14 females ADHD: *n* = 33 *M*age = 12.21 11 females Control: *n* = 32 *M*age = 12.13 18 females	Pre- and post-exclusion Positive And Negative Affect Schedule for Children (PANAS-C) showed exclusion increased negative mood only for the LOC eating group compared to the other groups, suggesting that ADHD in adolescents is not associated with enhanced negative emotional responses following social exclusion Impulsivity measurements for the SST showed that exclusion caused a difference in impulsivity between the ADHD and LOC eating groups, with a post-exclusion increase in the LOC group and a decrease in the ADHD group No correlation between mood (PANAS-C) and SST (impulsivity)	
Jankowski et al. (2018) [57] USA Cyberball Cyberball practice (baseline) vs. Inclusion via Cyberball vs. Exclusion via Cyberball vs. Short inclusion via Cyberball	Depression: *n* = 87 *M*age = 14.89 50 females Control: *n* = 39 *M*age = 14.43 20 females	fMRI exclusion > inclusion showed activation of medial prefrontal cortex / perigenual anterior cingulate cortex, left inferior frontal gyrus, right inferior frontal gyrus, right precentral gyrus, right postcentral gyrus, right superior temporal gyrus / middle temporal gyrus, and bilateral occipital cortex Depressive variables were correlated with neural patters during social exclusion (e.g., greater left middle temporal gyrus activity was positively correlated with self-worth)	fMRI exclusion > inclusion group contrasts showed that adolescents with depression had greater activity in the left anterior insula / inferior frontal gyrus compared to HC, and that HC had greater activity in the left middle temporal gyrus compared to depressed adolescents; anterior insula hypersensitivity may be related to heightened salience and an emotion regulation bias in participants with depression [70, 71]; differences in middle temporal gyrus activation may be related to altered emotion regulation in response to negative social information [91] Adolescents with depression recruited anterior insula / inferior frontal gyrus more during exclusion (and middle temporal gyrus to a similar degree) compared to inclusion; whereas HC recruited the anterior insula / inferior frontal gyrus more during inclusion compared to exclusion, and middle temporal gyrus more during exclusion; this corresponded to a significant difference between adolescents with depression and HC in middle temporal gyrus recruitment, but no significant group difference for the anterior insula / inferior frontal gyrus
Krauch et al. (2018) [65] Germany Script-driven imagery 8 scripts read by actors, each with 4 phases: 1. baseline 2. rejection-based anger 3. other-directed/self-directed aggression 4. relaxation	Adolescents with BPD: *n* = 20 *M*age = 16.35 20 females Adolescent control: *n* = 20 *M*age = 15.85 20 females Adults with BPD: *n* = 34 *M*ean age = 25.69 34 females Adult control: *n* = 32 *M*age = 27.33 32 females	Self-reported measures showed that adolescents with BPD did not differ in their reaction to rejection-based anger compared to HC, but did report higher dissociation fMRI showed that during rejection-based anger, adolescents with BPD had higher activity in in a large cluster comprising parts of the left insula, putamen, and claustrum, compared to HC; suggesting that early developmental stages of BPD are associated with an enhanced neural reactivity to rejection-related anger; these findings highlight the need of appropriate early interventions for adolescents with BPD	
Latina et al. (2023) [66] Germany Cyberball + WhatsApp chat simulation (SOLO) Inclusion via Cyberball vs. Exclusion via Cyberball Inclusion via SOLO vs. Exclusion + name calling + mobbing via SOLO	Emotion dysregulation: *n* = 23 *M*ean age = 14.74 17 females Control: *n* = 12 *M*age = 16.0 5 females	Self-reported emotional affect was measured before and after each paradigm, but the reported results only include SOLO vs. Cyberball paradigm comparisons (i.e., not between the conditions of each paradigm) Reported heart rate (HR) and heart rate variability (HRV) were measured throughout the experiment, but the reported results only include paradigm comparisons	Self-reported emotional affect results showed that adolescents in the emotion dysregulation group, but not healthy adolescents, had higher negative affect after the SOLO paradigm compared to Cyberball HR and HRV results showed that adolescents in the emotion dysregulation group, but not healthy adolescents, had higher HR and lower HRV during SOLO compared to Cyberball
Masten et al. (2011) [64] USA Cyberball Inclusion via Cyberball vs. Exclusion via Cyberball	Autism spectrum condition (ASC): *n* = 19 *M*age = 14.0 1 female Control: *n* = 17 *M*age = 13.6 2 females	Self-reported feelings of distress using NTS showed moderate levels of social distress following social exclusion for both groups fMRI exclusion > inclusion showed higher activation in the subgenual anterior cingulate cortex, anterior insula, ventrolateral prefrontal cortex, and ventral striatum for both groups	fMRI exclusion > inclusion contrasts showed that adolescents with ASC had less activation compared to neurotypical adolescents in the subgenual anterior cingulate cortex and anterior insula, areas linked to more distress caused by social exclusion; and at the same time less activation in the ventrolateral prefrontal cortex, an area linked to the regulation of distress caused by social exclusion Although those with ASC showed less neural activity in brain regions previously linked to distress and distress regulation during peer exclusion [6, 73], both groups were equally aware and concerned (i.e., self-reported distress) about peer rejection
Mellick (2017) [61] USA Cyberball Inclusion via Cyberball vs. Exclusion via Cyberball	Depression: *n* = 17 *M*age = 15.53 13 females Control: *n* = 18 *M*age = 14.11 10 females	fMRI exclusion > inclusion contrasts showed that adolescents with depression had higher activation in the right anterior insula, left occipital operculum (this is mentioned in abstract and discussion, but not in the results), and left nucleus accumbens compared to HC fMRI inclusion > exclusion contrasts showed that HC had higher activation in the right precuneus and right middle cingulate gyrus compared to adolescents with depression	
Müller et al. (2017) [62] Germany Emotion recognition task (ERT) + Cyberball ERT 1 week before (baseline) vs. ERT 1 week later after: Inclusion via Cyberball Exclusion via Cyberball	High depressive symptoms (HD): *n* = 26 *M*age = 13.42 15 females Low depressive symptoms (LD): *n* = 34 *M*age = 13.25 25 females	No HC group ERT results at baseline did not indicate a difference between LD and HD adolescents LD and HD adolescents’ sensitivity to happy facial expressions differed depending on the role of the person during Cyberball (i.e., includer vs. excluder): LD adolescents were more sensitive to the happy faces of includers and strangers; HD adolescents were more sensitive to the happy faces of excluders, suggesting that depressive symptom severity alters post-social exclusion facial expression sensitivity	
Sebastian et al. (2009) [56] UK Cyberball Inclusion via Cyberball vs. Exclusion via Cyberball	ASC: *n* = 13 *M*age = 16.9 0 females Control: *n* = 13 *M*age = 16.9 0 females	NTS and related mood questions showed that social exclusion negatively affected both groups (compared to baseline and inclusion) State / Trait Anxiety Inventory (STAI-S / STAI-T) showed that state anxiety was lower for both groups after inclusion (compared to baseline and exclusion)	For the NTS, post-ostracism meaningful existence ratings decreased more for adolescents with ASC than for neurotypical controls The mood section of the NTS showed that in neurotypical adolescents, but not in adolescents with ASC, following social exclusion the mood decreased compared to the baseline and inclusion
Silk et al. (2014) [63] USA Chatroom Interact Task (CIT) Participants choose to interact / exclude virtual peers 2 weeks before (baseline) vs. 2 weeks later participants are told that they were included / excluded from an interaction with virtual peers	Major depressive disorder: *n* = 21 Control: *n* = 27 Mean age and gender were not described for each sub-sample, but rather for the whole sample: *N* = 48 *M*age = 15.48 34 females	fMRI showed that adolescents in a more advanced pubertal status had higher activation to social exclusion in both right and left amygdala / parahippocampal gyrus, and the caudate / subgenual anterior cingulate cortex fMRI results showed that following inclusion, the groups did not differ in neural activation	Post-CIT self-reported feelings showed that adolescents with a major depressive disorder reported being more “sad”, “nervous” and “excluded”, and less “happy” compared to HC; groups did not differ in feeling “included” fMRI showed that adolescents with a major depressive disorder had greater activity in the bilateral amygdala, subgenual anterior cingulate cortex, left anterior insula, and left nucleus accumbens compared to HC; supporting the notion that neural reactivity to peer rejection seems to be particularly enhanced in youth with depression

Note. Included outcomes only relate to social exclusion and to adolescent participants. Outcomes related to interaction effects were merged to appear in both outcome columns

### Quality assessment

A quality assessment to evaluate the risk of bias in individual studies was done using the Newcastle-Ottawa scale [68]. This scale evaluates the quality of non-randomized studies using a star system, with a higher number of stars indicating a higher assessment of quality (for more details about the scale, please see the appended protocol). Notably, none of the included experiments received a star for case representativeness. However, this could be deemed reasonable, as the samples included clinical populations, and participants were most likely inpatients in the various institutes the experimenters were affiliated with. Most experiments scored high scores (i.e., between five to seven stars out of a total of nine), indicating a lower risk of bias. Two experiments scored lower scores (i.e., three or four stars), indicating a higher risk of bias [65, 66]. In these experiments, there was no adequate case definition. Moreover, the control groups’ selection and definition were inadequate. Additionally, in Latina et al. [66], comparability with the clinical group was inadequate. Nevertheless, all studies fulfilled the inclusion criteria. For a summary of the quality assessment across each of the studies please see Table 2.

**Table 2:** Quality assessment

	Brown et al. (2017) [59]	Gifuni et al. (2024) [47]	Groschwitz et al. (2016) [60]	Hartmann et al. (2013) [58]	Jankowski et al. (2018) [57]	Krauch et al. (2018) [65]	Latina et al. (2023) [66]	Masten et al. (2011) [64]	Mellick (2017) [61]	Müller et al. (2017) [62]	Sebastian et al. (2009) [56]	Silk et al. (2014) [63]
Is the case definition adequate?	★	-	★	★	-	-	-	★	★	★	★	★
Representativeness of the cases	-	-	-	-	-	-	-	-	-	-	-	-
Selection of controls	-	★	-	-	★	-	-	★	★	★	-	★
Definition of controls	-	-	★	-	-	-	-	★	-	-	★	-
Comparability of cases and controls based on the design or analysis	★★	★★	★★	★★	★★	★	-	★	★★	-	★★	★★
Ascertainment of exposure	★	★	★	★	★	★	★	★	★	★	★	★
Same method of ascertainment for cases and controls	★	★	★	★	★	★	★	★	★	★	★	★
Non-Response rate	★	★	★	★	★	★	★	★	★	★	★	★

Note. Based on the Newcastle-Ottawa scale [68]. “★” corresponds with low risk of bias and “-” corresponds with high risk of bias

### Results of individual studies

#### General effects of social exclusion

For self-report measures, most studies found a negative association between social exclusion and scores on questionnaires such as the NTS [56, 60, 64]. This was also the case with the Positive And Negative Affect Schedule for Children (PANAS-C) [58], and the anxiety measures State / Trait Anxiety Inventory (STAI-S / STAI-T) [56]. However, several studies only administered these measures post-manipulation and compared groups, making it impossible to conclude whether group differences were pre-existing or resulted from groups’ differential sensitivity to exclusion (e.g., [47, 59]). Additionally, one study did not find an association between social exclusion and self-report measures [65], but that might be due to the used paradigm (i.e., script-driven imagery). In this paradigm, participants had to listen to a script and imagine the described scenes as vividly as possible. Thus, the extent of experienced social exclusion depends on the power of imagination of the individual. Therefore, it is possible that due to the nature of the task, social exclusion was not experienced as vividly as it would have had the researchers used another paradigm (e.g., Cyberball). Moreover, one study only compared two different exclusion paradigms [66]. This made it impossible to deduce the separate impact of each paradigm. In this study, heart rate and heart rate variability were assessed, but only the relative effect of two exclusion paradigms was reported [66]. Another study, that did not have a healthy control group but rather adolescents with low or high depressive symptoms, showed an effect using an emotion recognition task [62].

The eight studies using fMRI reported various contrasts (inclusion > exclusion, inclusion > baseline / observation), which showed activation in various brain areas. Several of these areas have previously been associated with social exclusion, such as the insula (anterior: [60, 61, 64]; left: [47]), anterior cingulate cortex ([60]; pregenual / dorsal: [59]; perigenual: [57]; subgenual: [64]), prefrontal cortex (ventrolateral / dorsolateral: [59, 64]; medial: [57]), ventral striatum [59, 69], and inferior frontal gyrus (left / right: [47, 57]). Moreover, Gifuni et al. [47] found an association between lower insula activation and lower feeling of belongingness, indicating that activity in this brain region might play a key role in establishing the feeling of “being connected” with others.

#### Adolescents with depression

Results among the studies that focused on the impact of social exclusion on adolescents with depression varied. Results from behavioral measures were ambiguous: whereas the post-exclusion NTS indicated higher distress levels for the clinical group in one study [47], in other studies [59, 60] the NTS showed no such effects. Silk et al. [63] showed that the clinical group reported being more “sad”, “nervous” and “excluded”, and less “happy” compared to healthy adolescents. Similarly, Müller et al. [62] showed that adolescents with depression identify emotions in ambiguous faces differently depending on their symptoms’ severity. Specifically, they showed an interaction effect, wherein “high depression” adolescents exhibited the highest perceptual sensitivity to happy faces depicted by an excluder, compared to includer and stranger models, the inverse pattern of “low depression” adolescents. The fMRI group contrasts showed that compared with healthy controls, adolescents with depression had higher exclusion-related neural activation in the insula [47, 57, 61, 63], subgenual anterior cingulate [63], putamen [59], left occipital operculum [61], and inferior frontal gyrus [57]. Hypersensitivity of this brain network may be related to enhanced salience and an emotion regulation bias in adolescents with depression [70, 71]. Moreover, the addition of NSSI led to prefrontal cortex activation [59, 60], but conversely *lower* exclusion-related activation in the inferior frontal gyrus (and right middle / superior frontal gyrus) reported by Gifuni et al. [47], who also reported lower precuneus activation, as did Mellick [61]. The prefrontal cortex plays a crucial role in emotion regulation [72]; modulations of activity in these brain circuits might indicate altered neural processing of social exclusion that is related to the absence or presence of NSSI [60].

In sum, studies focusing on adolescents with depression found that social exclusion impacted them differently than inclusion, and found some differences in the impact on them compared to healthy adolescents, particularly in the insula.

#### Adolescents with autism spectrum condition (ASC)

Both studies that focused on adolescents with ASC found that they were impacted by social exclusion. The results from behavioral measures (i.e., NTS, STAI-S) obtained by Sebastian et al. [56] showed that adolescents both with and without ASC self-reported more negative needs and anxiety following social exclusion compared to baseline and inclusion. Conversely, they showed that neurotypical adolescents, but *not* adolescents with ASC, had a decreased mood following social exclusion. In contrast, Masten et al. [64] found no group differences using the NTS. However, their fMRI results showed that compared to neurotypical adolescents, adolescents with ASC had less neural activation in the subgenual anterior cingulate cortex, anterior insula, ventrolateral prefrontal cortex, and ventral striatum following social exclusion vs. inclusion; thus, individuals with ASC showed less neural activity in brain regions that have previously been associated with distress and distress regulation during social exclusion [6, 73]. This finding is in line with previous research showing that individuals with ASC show hypoactivation in brain regions that have been linked to emotion processing [74].

In sum, as with depression, studies focusing on adolescents with ASC found that social exclusion impacted them differently than inclusion, and found some differences in the impact on them compared to healthy adolescents.

#### Adolescents with other psychiatric disorders

Results among the studies that focused on the impact of social exclusion on adolescents with other disorders varied considerably. In terms of social exclusion’s impact, the results obtained by Hartmann et al. [58] provide partial support to the impact of social exclusion. Moreover, the results obtained by Krauch et al. [65] and Latina et al. [66] provide no support or are not reported. Specifically, Hartmann et al. [58] compared adolescents with loss of control eating or ADHD with healthy adolescents. They found an interaction effect for group by time in impulsivity (i.e., impulsivity increased with time) but not in self-reported affect following social exclusion. Krauch et al. [65] focused on adolescents with BPD and found that self-reported measures (e.g., subjective anger ratings) were not affected by social exclusion. However, they induced social exclusion via script-driven imagery, which might be less powerful than other paradigms such as Cyberball. Furthermore, although fMRI was measured throughout the experiment, the conditions’ neurophysiological impact is unknown as they only reported results concerning group comparisons. Latina et al. [66] focused on adolescents with various psychiatric disorders (i.e., grouped under the umbrella term “emotional dysregulation”). Similar to Krauch et al. [65], they too only reported results concerning group comparisons. In terms of group differences, Hartmann et al. [58] found no differences between adolescents with loss of control eating or ADHD and healthy adolescents. Krauch et al. [65] found some self-reported differences between adolescents with BPD and healthy adolescents (i.e., higher dissociation), as well as differences in neural activation. Latina et al. [66] found both self-reported and psychophysiological differences between adolescents with emotional dysregulation and healthy adolescents. However, their focus was not on the impact of social exclusion, but rather on the comparison between different social exclusion paradigms (i.e., SOLO vs. Cyberball). Moreover, it is possible that these paradigms were not comparable since the social exclusion phase in SOLO includes additional cyberbullying (i.e., which the authors address briefly as name calling and mobbing).

In sum, based on these studies, the impact of social exclusion on adolescents with other disorders was inconclusive and to some extent remains unknown.

## Discussion

### Summary of evidence

The aim of the current study was to systematically review experimental investigations of social exclusion among adolescents with psychiatric disorders. This systematic review was done in accordance with the PRISMA framework [45]. Twelve experiments that met pre-defined inclusion criteria were included. Although the results from these experiments were partly inconclusive, a certain pattern can be deduced. Specifically, both adolescents with and without psychiatric disorders are impacted by social exclusion. Moreover, fMRI measurements provide evidence to an altered neural reaction in adolescents with psychiatric disorders in response to social exclusion. Notably, the included experiments varied considerably in terms of clinical sample characteristics, methodology, and reported results. For a summary of the critical findings please see Table 3.

**Table 3:** Summary of the systematic review’s critical findings

Critical findings
• 12 experiments investigating social exclusion among adolescents with psychiatric disorders were included
• All 12 showed that social exclusion impacts adolescents with psychiatric disorders differently than inclusion
• The most researched psychiatric disorder was depression, featured in 58% (*n* = 7) of the experiments
• 42% (*n* = 5) of the experiments found a conclusive group difference in clinical vs. healthy controls using both behavioral measurements and neuroimaging (i.e., fMRI). All experiments containing fMRI measurements revealed group differences in brain activation, pointing toward altered neural responding in adolescents with psychiatric disorders (e.g., heightened neural reactivity to social exclusion in adolescents with depression)
• Cyberball was the most used paradigm, used in 83% (*n* = 10) of the experiments

### Social exclusion and psychiatric disorders

Among the included experiments, the most researched psychiatric disorder was depression (*n* = 7), followed by various other disorders (*n* = 3) and ASC (*n* = 2; please find detailed information in the “Results of individual studies” sub-section). Evidence from the experiments researching both adolescents with depression and adolescents with ASC indicates that social exclusion impacts them differently than inclusion does. Evidence from the experiments researching adolescents with various other disorders (e.g., ADHD) varied considerably. Although there was some support to a different impact of the condition (i.e., social exclusion vs. inclusion), and to group differences (i.e., clinical vs. control group), it is difficult to draw clear conclusions. This difficulty arises from these studies’ choice of paradigms (e.g., listening to a script, which might not be powerful enough to elicit social exclusion), and from missing results (i.e., unknown comparisons). Thus, more research is needed investigating a sample with a broad range of disorders in a unified transparent manner. Please see Table 4 for a recommended checklist for future research, as well as implications for clinicians and policy.

ASC is a particularly interesting condition in the context of social exclusion. Namely because individuals with ASC often struggle in social situations with neurotypicals [75]. Interestingly, Sebastian et al. [56] found that after social exclusion, typically developing adolescents, but not adolescents with ASC, had a decreased mood. This stood out from the other experiments included in this review that either showed no difference, or an increased response in clinical populations compared to healthy adolescents. One of the possible reasons for this difference that Sebastian et al. [56] suggested, is the difficulty many adolescents with ASC have in interpreting their own emotional state. Another possibility is that adolescents with ASC struggle with perceiving social exclusion [76]. To examine this, the study by Sebastian et al. [56] could be replicated with an additional measurement of how the situation is being perceived (e.g., the understanding score used by Hodgins et al. [76]). This suggestion as well as further research with adolescents with ASC could contribute valuable insights.

Moreover, other disorders such as antisocial personality disorder (ASPD) would be a great avenue for future research. ASPD could be particularly interesting in the context of social exclusion, as among other things it is characterized by failure to conform with social norms and a tendency to react aggressively when angry [77]. Notably, ASPD is not diagnosable before the age of 18. This means that there is only a one-year time frame left to test adolescents with ASPD (i.e., before they turn 19; i.e., according to the age range for adolescence defined by the World Health Organization [46]). To combat this challenge, adolescents diagnosed with conduct disorder before the age of 15, a pre-requisite for an ASPD diagnosis, could be contacted and potentially recruited in time. One interesting experiment that was excluded from the current systematic review (i.e., since it included older participants, and there was no clinical diagnosis but rather a measurement of traits), examined the impact of psychopathic traits on responses to social exclusion [49]. One of their findings was that participants high on antisocial traits were angrier after being socially excluded. Furthermore, adolescents with psychiatric comorbidity were examined only in some of the included experiments (e.g., adolescents with depression and NSSI; [59]). Although psychiatric comorbidity is widely common in developing populations, to date, it is still common practice for clinical research to focus on isolated disorders and exclude participants with additional deficits [78]. Thus, research examining other disorders such as ASPD, as well as research looking into psychiatric comorbidity, could be highly beneficial.

### Impact of social exclusion on clinical vs. healthy control (HC) samples

Evidence regarding group differences between clinical populations and HC was inconclusive. Specifically, fMRI data showed group differences, but support from behavioral measures was inconsistent. Therefore, it is possible that group differences are most prominent on a neurophysiological level, pointing toward sensitization processes in the brain. According to previous meta-analyses and reviews, the brain regions predominantly related to social exclusion are: the posterior cingulate cortex, posterior insula, anterior insula, anterior cingulate cortex, prefrontal cortex, temporal cortex, precuneus, ventral striatum, inferior gyrus and superior frontal gyrus, and the occipital pole [79–81]. Future studies implementing fMRI should investigate all the above-mentioned brain regions (e.g., by using appropriate regions of interest analyses). Moreover, when using behavioral measures, baseline measurements should be conducted to clearly distinguish between differences related to psychiatric disorders from those caused by social exclusion.

### Social exclusion paradigms

Among the included experiments, Cyberball [42] was the most commonly used paradigm (*n* = 10). This is not surprising, since Cyberball is free to use, easy to implement, and has been well-established in research since its early versions for more than two decades. Multiple other social exclusion paradigms have been developed over the years, including: Get Acquainted [82], Life Alone [1], O-Cam [83], Ostracism Online [84], and social media vignettes [85]. It is possible that these paradigms were not tested on developing clinical populations as the experimenters did not have access to patients in medical institutions. In turn, experimenters with access to patients in medical institutions might have not used these paradigms as they preferred to use Cyberball [42], knowing it is so well-established. One paradigm that has been tested on a developing clinical population is SOLO, developed by Latina et al. [66]. SOLO has high ecological validity, since it simulates being socially rejected in a chat on WhatsApp, a communication platform widely used by adolescents. Although Latina et al. [66] found that SOLO leads to more negative effects than Cyberball, their experiment included additional elements (e.g., name calling), which make the comparison problematic. Future studies using SOLO without these additional elements, or other ecologically valid paradigms, on a sample of adolescents with psychiatric disorders could contribute valuable insights. Please see Table 4 for the implications of the systematic review for practice and policy, and a checklist for future research.

**Table 4:** Implications of the systematic review for practice, policy, and checklist for future research

Implications for practice	Implications for policy	Recommended checklist for future research
When treating adolescents with psychiatric disorders, practitioners should evaluate if they are being socially excluded If yes, practitioners should assist these adolescents in developing strategies to combat negative impacts on their mood and well-being If yes, special care should be taken to identify potential violent reactions to facilitate its prevention	Emphasize social exclusion’s negative impact, especially among adolescents with psychiatric disorders in new and existing programs (e.g., anti-bullying programs)	- Explore the impact of social exclusion on samples with various disorders (e.g., ASPD) and psychiatric comorbidity - Use fMRI to measure brain regions that have been linked to social exclusion by meta-analyses and reviews [79–81]: - posterior cingulate cortex - posterior insula - anterior insula - anterior cingulate cortex - prefrontal cortex - temporal cortex - precuneus - ventral striatum - inferior gyrus and superior frontal gyrus - occipital pole - Conduct appropriate baseline measurements - Test ecologically valid paradigms (e.g., SOLO without additional bullying elements)

### Limitations

This systematic review is not without its limitations. Firstly, both a meta-analysis (which would allow the computation of combined effect sizes) and a formal preregistration were not conducted. A meta-analysis was not feasible due to the different nature of the included experiments, which did not allow a comparison of effect sizes. Nevertheless, we believe that the grouping of the included experiments into three disorder groups allowed for some valuable comparability. Moreover, the systematic review was not preregistered on a public website. However, the researchers followed a pre-defined protocol adhering to the PRISMA statement [45], which is available in the Appendix. Secondly, all of the included experiments had a within-subject design with regard to the experimental manipulation (social exclusion). Moreover, only some included a comparison between social exclusion and a quasi-baseline condition (e.g., passively watching other participants playing Cyberball). This undermines their support to the possibility of causal links between psychiatric disorders and social exclusion [86, 87]. Thus, in some cases it was not possible to deduce the impact of social exclusion, but rather its association with the different measurements. One notable experiment we had to exclude (i.e., as its sample included adults) was by Meneguzzo et al. [52]. In this experiment, a between-subjects design was used to clearly distinguish the impact of social exclusion from that of inclusion on people with eating disorders. Thirdly, gender diversity was compromised, as gender was not equally distributed in the included experiments. In particular, experiments focused on adolescents with depression (e.g., [60]) had a predominantly female sample. Conversely, experiments focused on adolescents with ASC had a predominantly male sample (e.g., [64]). This is probably related to gender differences in the prevalence of different psychiatric disorders [88]. Nevertheless, future experiments should strive to recruit gender balanced samples. Fourthly, six experiments that mixed adolescents with other age groups were excluded, which might have compromised the findings. We reached out to these papers’ corresponding authors if a current email address was available (i.e., five out of the six), and asked if they performed separate analyses for adolescents. One author replied and said they did not, and the others were not responsive. Lastly, we chose a medical approach, which compromises neurodiversity. This was done to clearly distinguish clinically diagnosed samples from control samples, and to draw general comparisons between different disorders. To promote diversity when deemed possible, when describing autistic individuals, we used the term “condition” (i.e., autism spectrum condition and not disorder). This was done in accordance with the prevailing outlook that autism is a difference rather than a disorder [89].

## Conclusions

This paper systematically reviewed experimental investigations of social exclusion among adolescents with psychiatric disorders. The review revealed that social exclusion impacts adolescents with psychiatric disorders differently than inclusion, both neurophysiologically and behaviorally (e.g., eliciting a negative emotional state). The psychiatric disorder most included experiments focused on was depression. The difference in the impact of social exclusion on adolescents with vs. without psychiatric disorders was inconclusive. Namely, we found differences between patients and healthy participants with neurophysiological (i.e., fMRI) but not with behavioral measures. Thus, it is possible that group differences are related to altered neural sensitivity and can thus predominantly be observed on the level of brain activity. More research is needed exploring a wider range of disorders associated with social exclusion. Furthermore, more research including baseline measurements and ecologically valid paradigms would be highly beneficial. It is advisable that practitioners treating adolescents with psychiatric disorders screen for social exclusion, and act to prevent its negative impact and potential violent reactions. Lastly, it is recommended that policy makers emphasize social exclusion’s negative impact, especially among adolescents with psychiatric disorders, in new and existing anti-bullying programs.

## Electronic supplementary material

Below is the link to the electronic supplementary material.

Supplementary Material 1

## Data Availability

No datasets were generated or analysed during the current study.
