# Supplementary material for: Experimental investigations of social exclusion among adolescents with psychiatric disorders: a systematic review
Source: Eur Child Adolesc Psychiatry. 2025 Apr 30;34(9):2631–48. doi: 10.1007/s00787-025-02687-9 (PMC12508016; doi:10.1007/s00787-025-02687-9)
Supplement: Supplementary file 1 — Supplementary Material 1 [file 787_2025_2687_MOESM1_ESM.docx]

# **Appendix**

## Protocol for “Experimental Investigations of Social Exclusion Among Adolescents with Psychiatric Disorders: A Systematic Review”

### Aim

The aim of this paper is to systematically review experiments that induced social exclusion in adolescents with psychiatric disorders. The main focus is on the comparison of social exclusion outcomes regarding two aspects. The first aspect being the impact of the exclusion condition vs. the baseline. Whereas the second aspect is on the impact of the exclusion condition on a clinical vs. a control group. The secondary focus of this review is to compare included studies across: country; sample (i.e., disorder in the clinical group, *N*, mean age, gender); paradigm (i.e., paradigm(s) name(s), within/between-subjects design, conditions); and measures (i.e., behavioral and neurophysiological).

### Eligibility Criteria

The Eligibility criteria will be:

- Empirical studies written or translated to English
- Publication type: journal articles and dissertations
- Study type: any study design
- Method: social exclusion experimentally induced
- Outcome data: impact of social exclusion
- Sample:
  - Sample includes a clinical population of adolescents with an officially assessed psychiatric disorder
  - Adolescent in the age range of 10-19 (i.e., the age range for adolescence defined by the World Health Organization; WHO, 2021)
  - No geographical restriction

### Information Sources

To facilitate the search, the following databases will be used: PubMed, Web of Science, PsycInfo, ERIC, and Cochrane. Additionally, a Google Scholar search and a manual reference list search of included experiments will be done.

### Study Selection

The process of experiment selection will be mainly done by the first author. First a screening of all search results’ abstracts will be done in accordance with the eligibility criteria. Then, the full texts of the potentially relevant experiments will be read by the first and last authors. These authors will independently decide on the included experiments. If any disagreements arise, they will be resolved in a discussion with the other authors.

### Data Collection Process

Selected experiments will be summarized by the first author in a preliminary Excel table detailing: title, sample characteristics, methods, paradigms, key results, and comments. The last author will assess the table. If any disagreements arise, they will be resolved in a discussion with the other authors.

### Data Items

The preliminary Excel table containing summaries of the selected experiments will be edited by the first author taking into consideration the last author’s input.

### Risk of Bias in Individual Studies

The Newcastle-Ottawa scale (Wells et al., 2021) will be used by the first author to assess the risk of bias in each of the selected experiments. This scale is used to assess the quality of non-randomized experiments. The rated parameters are: sample selection, sub-sample comparability, and outcome assessment. The rating is done using a star system, with a maximum score of nine stars. A higher number of stars indicates a higher assessment of quality. The last author will assess the rating. If any disagreements arise, they will be resolved in a discussion with the other authors.

### Search Strategy

| *PubMed* | | |
| --- | --- | --- |
| Search | Search terms | Number of results |
| #1 | child* [tw] OR adolescen* [tw] OR kid* [tw] OR youth [tw] OR teenage* [tw] OR "youth mental health" [tw] OR "adolescent psychology" [tw] OR "child psychology" [tw] | 3,984,182 |
| #2 | Child [mh] OR Adolescent [mh] | 3,387,857 |
| #3 | cyberbully* [tw] OR "online ostraci*" [tw] OR "cyber ostraci*" [tw] OR "online bully*" [tw] OR "cyber victim*" [tw] OR "cyber aggress*" [tw] OR cyberperpetrat* [tw] OR cyberbystander* [tw] OR "cyberbullying bystander*" [tw] OR "cyber harassment" [tw] OR "cyber abuse" [tw] OR "cyber deviance" [tw] OR (bullying [tiab] AND ("social media" [tiab] OR internet [tiab] OR technology [tiab])) OR (ostracism [tiab] AND ("social media" [tiab] OR internet [tiab] OR technology [tiab])) OR (victimi* [tiab] AND ("social media" [tiab] OR internet [tiab] OR technology [tiab])) OR ("social exclusion" [tiab] AND ("social media" [tiab] OR internet [tiab] OR technology [tiab])) | 2,496 |
| #4 | Cyberbullying [mh] | 621 |
| #5 | paradigm* [tw] OR "experimental paradigm*" [tw] OR "experimental task*" [tw] OR Cyberball [tw] OR O-Cam [tw] OR Atimia [tw] OR ScrollQuest [tw] OR "chat room" [tw] OR "ostracism online" [tw] OR recall [tw] OR No1LikesU! [tw] OR vignette* [tw] OR "social media vignette*" [tw] | 283,237 |
| #6 | "psychiatric disorder*" [tw] OR "learning disorder*" [tw] OR "learning disab*" [tw] OR dyslexia [tw] OR acalculia [tw] OR adhd [tw] OR "attention deficit disorder with hyperactivity" [tw] OR anxiety [tw] OR "anxiety disorder*" [tw] OR "psychiatric comorbidit*" [tw] OR suicidality [tw] OR "suicidal ideation" [tw] OR anorexia [tw] OR "anorexia nervosa" OR "conduct disorder*" [tw] OR depression [tw] OR "depressive disorder*" [tw] OR "affective disorder*" [tw] OR "major depression" [tw] OR "persistent depressive disorder*" [tw] OR autism [tw] OR asperger [tw] OR "Oppositional Defiant Disorder" [tw] OR "intellectual disorder" [tw] OR "intellectual disability" [tw] OR disability [tw] OR "inclusive school" [tw] OR "special needs" [tw] OR "special education" [tw] OR neurodevelopment [tw] OR cognitive [tw] OR tourettes [tw] OR dyscalculia [tw] OR "borderline personality disorder" [tw] OR "autism spectrum disorder" [tw] OR "autistic disorder" [tw] OR "tourette syndrome" [tw] OR "asperger syndrome" [tw] OR "cognitive dysfunction" [tw] OR "intellectual disability" [tw] OR "attention deficit and disruptive behavior disorders" [tw] | 1,573,383 |
| #7 | "Mental Disorders" [mh] OR "Learning Disabilities" [mh] OR "Attention Deficit Disorder with Hyperactivity" [mh] OR Anxiety [mh] OR "Anxiety Disorders" [mh] OR "Suicidal Ideation" [mh] OR Anorexia [mh] OR "Anorexia Nervosa" [mh] OR "Conduct Disorder" [mh] OR Depression [mh] OR "Depressive Disorder" [mh] OR "Autism Spectrum Disorder" [mh] OR "Autistic Disorder" [mh] OR "Tourette Syndrome" [mh] OR "Asperger Syndrome" [mh] OR "Cognitive Dysfunction" [mh] OR "Intellectual Disability" [mh] OR "Attention Deficit and Disruptive Behavior Disorders" [mh] | 1,645,239 |
| #5 & #1 OR #2 & #3 OR #4 & #6 OR #7 | ("paradigm*"[Text Word] OR "experimental paradigm*"[Text Word] OR "experimental task*"[Text Word] OR "Cyberball"[Text Word] OR "O-Cam"[Text Word] OR "Atimia"[Text Word] OR "chat room"[Text Word] OR "ostracism online"[Text Word] OR "recall"[Text Word] OR "No1LikesU"[Text Word] OR "vignette*"[Text Word]) AND ("child*"[Text Word] OR "adolescen*"[Text Word] OR "kid"[Text Word] OR "youth"[Text Word] OR "teenage*"[Text Word] OR "youth mental health"[Text Word] OR "adolescent psychology"[Text Word] OR "child psychology"[Text Word] OR ("child"[MeSH Terms] OR "adolescent"[MeSH Terms])) AND ("cyberbully*"[Text Word] OR "online ostraci*"[Text Word] OR "cyber ostraci*"[Text Word] OR "online bully*"[Text Word] OR "cyber victim*"[Text Word] OR "cyber aggress*"[Text Word] OR "cyberperpetrat*"[Text Word] OR "cyberbystander*"[Text Word] OR "cyberbullying bystander*"[Text Word] OR "cyber harassment"[Text Word] OR "cyber abuse"[Text Word] OR "cyber deviance"[Text Word] OR ("bullying"[Title/Abstract] AND ("social media"[Title/Abstract] OR "internet"[Title/Abstract] OR "technology"[Title/Abstract])) OR ("ostracism"[Title/Abstract] AND ("social media"[Title/Abstract] OR "internet"[Title/Abstract] OR "technology"[Title/Abstract])) OR ("victimi*"[Title/Abstract] AND ("social media"[Title/Abstract] OR "internet"[Title/Abstract] OR "technology"[Title/Abstract])) OR ("social exclusion"[Title/Abstract] AND ("social media"[Title/Abstract] OR "internet"[Title/Abstract] OR "technology"[Title/Abstract])) OR "cyberbullying"[MeSH Terms]) AND ("psychiatric disorder*"[Text Word] OR "learning disorder*"[Text Word] OR "learning disab*"[Text Word] OR "dyslexia"[Text Word] OR "acalculia"[Text Word] OR "adhd"[Text Word] OR "Attention Deficit Disorder with Hyperactivity"[Text Word] OR "anxiety"[Text Word] OR "anxiety disorder*"[Text Word] OR "psychiatric comorbidit*"[Text Word] OR "suicidality"[Text Word] OR "Suicidal Ideation"[Text Word] OR "anorexia"[Text Word] OR "Anorexia Nervosa"[All Fields] OR "conduct disorder*"[Text Word] OR "depression"[Text Word] OR "depressive disorder*"[Text Word] OR "affective disorder*"[Text Word] OR "major depression"[Text Word] OR "persistent depressive disorder*"[Text Word] OR "autism"[Text Word] OR "asperger"[Text Word] OR "Oppositional Defiant Disorder"[Text Word] OR "intellectual disorder"[Text Word] OR "Intellectual Disability"[Text Word] OR "disability"[Text Word] OR "inclusive school"[Text Word] OR "special needs"[Text Word] OR "special education"[Text Word] OR "neurodevelopment"[Text Word] OR "cognitive"[Text Word] OR "tourettes"[Text Word] OR "dyscalculia"[Text Word] OR "borderline personality disorder"[Text Word] OR "Autism Spectrum Disorder"[Text Word] OR "Autistic Disorder"[Text Word] OR "Tourette Syndrome"[Text Word] OR "Asperger Syndrome"[Text Word] OR "Cognitive Dysfunction"[Text Word] OR "Intellectual Disability"[Text Word] OR "Attention Deficit and Disruptive Behavior Disorders"[Text Word] OR ("Mental Disorders"[MeSH Terms] OR "Learning Disabilities"[MeSH Terms] OR "Attention Deficit Disorder with Hyperactivity"[MeSH Terms] OR "anxiety"[MeSH Terms] OR "Anxiety Disorders"[MeSH Terms] OR "Suicidal Ideation"[MeSH Terms] OR "anorexia"[MeSH Terms] OR "Anorexia Nervosa"[MeSH Terms] OR "Conduct Disorder"[MeSH Terms] OR ("Depressive Disorder"[MeSH Terms] OR "depression"[MeSH Terms]) OR "Depressive Disorder"[MeSH Terms] OR "Autism Spectrum Disorder"[MeSH Terms] OR "Autistic Disorder"[MeSH Terms] OR "Tourette Syndrome"[MeSH Terms] OR "Asperger Syndrome"[MeSH Terms] OR "Cognitive Dysfunction"[MeSH Terms] OR "Intellectual Disability"[MeSH Terms] OR "Attention Deficit and Disruptive Behavior Disorders"[MeSH Terms])) | 11 |

| *PsycInfo* | | |
| --- | --- | --- |
| Search | Search terms | Number of results |
| #1 | TX (child* OR adolescen* OR kid* OR youth OR teenage* OR "youth mental health" OR "adolescent psychology" OR "child psychology") | 1,420,461 |
| #2 | DE "Youth Mental Health" OR DE "Adolescent Psychology" OR DE "Child Psychology" | 10,829 |
| #3 | TX (cyberbully* OR "online ostraci*" OR "cyber ostraci*" OR "online bully*" OR "cyber victim*" OR "cyber aggress*" OR cyberperpetrat* OR cyberbystander* OR "cyberbullying bystander*" OR "cyber harassment" OR "cyber abuse" OR "cyber deviance") OR TX (bullying AND ("social media" OR internet OR technology)) OR TX (ostracism AND ("social media" OR internet OR technology)) OR TX (victimi* AND ("social media" OR internet OR technology)) OR TX ("social exclusion" AND ("social media" OR internet OR technology)) | 8,082 |
| #4 | DE Cyberbullying | 2,588 |
| #5 | TX (paradigm* OR "experimental paradigm*“ OR "experimental task*" OR Cyberball OR O-Cam OR Atimia OR ScrollQuest OR "chat room“ OR "ostracism online" OR recall OR No1LikesU! OR vignette* OR "social media vignette*") | 205,681 |
| #6 | TX ("psychiatric disorder*" OR "learning disorder*" OR "learning disab*" OR dyslexia OR acalculia OR adhd OR "attention deficit disorder with hyperactivity" OR anxiety OR "anxiety disorder*" OR "psychiatric comorbidit*" OR suicidality OR "suicidal ideation" OR anorexia OR "anorexia nervosa" OR "conduct disorder*" OR depression OR "depressive disorder*" OR "affective disorder*" OR "major depression" OR "persistent depressive disorder*" OR autism OR asperger OR "Oppositional Defiant Disorder" OR "intellectual disorder" OR "intellectual disability" OR disability OR "inclusive school" OR "special needs" OR "special education" OR neurodevelopment OR cognitive OR tourettes OR dyscalculia OR "borderline personality disorder" OR "autism spectrum disorder" OR "autistic disorder" OR "tourette syndrome" OR "asperger syndrome" OR "cognitive dysfunction" OR "intellectual disability" OR "attention deficit and disruptive behavior disorders") | 1,507,287 |
| #7 | DE "Mental Disorders" OR DE "Affective Disorders" OR DE "Learning Disabilities" OR DE "Attention Deficit Disorder with Hyperactivity" OR DE Anxiety OR DE "Anxiety Disorders" OR DE "Suicidal Ideation" OR DE Suicidality OR DE "Anorexia Nervosa" OR DE "Conduct Disorder" OR DE "Major Depression“ OR DE "Persistent Depressive Disorder" OR DE "Autism Spectrum Disorders" OR DE "Tourette Syndrome" OR DE "Cognitive Impairment" OR DE "Special Education Students" OR DE "Special Needs" OR DE "Borderline Personality Disorder" OR DE "Oppositional Defiant Disorder" | 550,695 |
| #5 & #1 OR #2 & #3 OR #4 & #6 OR #7 | (TX (paradigm* OR "experimental paradigm*“ OR "experimental task*" OR Cyberball OR O-Cam OR Atimia OR ScrollQuest OR "chat room“ OR "ostracism online" OR recall OR No1LikesU! OR vignette* OR "social media vignette*")) AND (TX (child* OR adolescen* OR kid* OR youth OR teenage* OR "youth mental health" OR "adolescent psychology" OR "child psychology") OR DE "Youth Mental Health" OR DE "Adolescent Psychology" OR DE "Child Psychology") AND (TX (cyberbully* OR "online ostraci*" OR "cyber ostraci*" OR "online bully*" OR "cyber victim*" OR "cyber aggress*" OR cyberperpetrat* OR cyberbystander* OR "cyberbullying bystander*" OR "cyber harassment" OR "cyber abuse" OR "cyber deviance") OR TX (bullying AND ("social media" OR internet OR technology)) OR TX (ostracism AND ("social media" OR internet OR technology)) OR TX (victimi* AND ("social media" OR internet OR technology)) OR TX ("social exclusion" AND ("social media" OR internet OR technology)) OR DE Cyberbullying) AND (TX ("psychiatric disorder*" OR "learning disorder*" OR "learning disab*" OR dyslexia OR acalculia OR adhd OR "attention deficit disorder with hyperactivity" OR anxiety OR "anxiety disorder*" OR "psychiatric comorbidit*" OR suicidality OR "suicidal ideation" OR anorexia OR "anorexia nervosa" OR "conduct disorder*" OR depression OR "depressive disorder*" OR "affective disorder*" OR "major depression" OR "persistent depressive disorder*" OR autism OR asperger OR "Oppositional Defiant Disorder" OR "intellectual disorder" OR "intellectual disability" OR disability OR "inclusive school" OR "special needs" OR "special education" OR neurodevelopment OR cognitive OR tourettes OR dyscalculia OR "borderline personality disorder" OR "autism spectrum disorder" OR "autistic disorder" OR "tourette syndrome" OR "asperger syndrome" OR "cognitive dysfunction" OR "intellectual disability" OR "attention deficit and disruptive behavior disorders") OR DE "Mental Disorders" OR DE "Affective Disorders" OR DE "Learning Disabilities" OR DE "Attention Deficit Disorder with Hyperactivity" OR DE Anxiety OR DE "Anxiety Disorders" OR DE "Suicidal Ideation" OR DE Suicidality OR DE "Anorexia Nervosa" OR DE "Conduct Disorder" OR DE "Major Depression“ OR DE "Persistent Depressive Disorder" OR DE "Autism Spectrum Disorders" OR DE "Tourette Syndrome" OR DE "Cognitive Impairment" OR DE "Special Education Students" OR DE "Special Needs" OR DE "Borderline Personality Disorder" OR DE "Oppositional Defiant Disorder") | 70 |

| *ERIC* | | |
| --- | --- | --- |
| Search | Search terms | Number of results |
| #1 | TX (child* OR adolescen* OR kid* OR youth OR teenage* OR "youth mental health" OR "adolescent psychology" OR "child psychology") | 479,569 |
| #2 | DE Children OR DE "Child Psychology" OR DE Adolescents | 96,077 |
| #3 | TX (cyberbully* OR "online ostraci*" OR "cyber ostraci*" OR "online bully*" OR "cyber victim*" OR "cyber aggress*" OR cyberperpetrat* OR cyberbystander* OR "cyberbullying bystander*" OR "cyber harassment" OR "cyber abuse" OR "cyber deviance") OR TX (bullying AND ("social media" OR internet OR technology)) OR TX (ostracism AND ("social media" OR internet OR technology)) OR TX (victimi* AND ("social media" OR internet OR technology)) OR TX ("social exclusion" AND ("social media" OR internet OR technology)) | 1,415 |
| #4 | DE Bullying | 6,249 |
| #5 | TX (paradigm* OR "experimental paradigm*" OR "experimental task*" OR Cyberball OR O-Cam OR Atimia OR ScrollQuest OR "chat room" OR "ostracism online" OR recall OR No1LikesU! OR vignette*OR "social media vignette*") | 33,372 |
| #6 | TX ("psychiatric disorder*" OR "learning disorder*" OR "learning disab*" OR dyslexia OR acalculia OR adhd OR "attention deficit disorder with hyperactivity" OR anxiety OR "anxiety disorder*" OR "psychiatric comorbidit*" OR suicidality OR "suicidal ideation" OR anorexia OR "anorexia nervosa" OR "conduct disorder*" OR depression OR "depressive disorder*" OR "affective disorder*" OR "major depression" OR "persistent depressive disorder*" OR autism OR asperger OR "Oppositional Defiant Disorder" OR "intellectual disorder" OR "intellectual disability" OR disability OR "inclusive school" OR "special needs" OR "special education" OR neurodevelopment OR cognitive OR tourettes OR dyscalculia OR "borderline personality disorder" OR "autism spectrum disorder" OR "autistic disorder" OR "tourette syndrome" OR "asperger syndrome" OR "cognitive dysfunction" OR "intellectual disability" OR "attention deficit and disruptive behavior disorders") | 292,218 |
| #7 | DE "Mental Disorders" OR DE "Learning Disabilities" OR DE "Attention Deficit Disorders" OR DE "Anxiety Disorders" OR DE Suicide OR DE "Depression (Psychology)" OR DE "Autism Spectrum Disoders" OR DE "Intellectual Disability" OR DE "Special Education" | 68,296 |
| #5 & #1 OR #2 & #3 OR #4 & #6 OR #7 | (TX (paradigm* OR "experimental paradigm*" OR "experimental task*" OR Cyberball OR O-Cam OR Atimia OR ScrollQuest OR "chat room" OR "ostracism online" OR recall OR No1LikesU! OR vignette*OR "social media vignette*")) AND (TX (child* OR adolescen* OR kid* OR youth OR teenage* OR "youth mental health" OR "adolescent psychology" OR "child psychology") OR DE Children OR DE "Child Psychology" OR DE Adolescents) AND (TX (cyberbully* OR "online ostraci*" OR "cyber ostraci*" OR "online bully*" OR "cyber victim*" OR "cyber aggress*" OR cyberperpetrat* OR cyberbystander* OR "cyberbullying bystander*" OR "cyber harassment" OR "cyber abuse" OR "cyber deviance") OR TX (bullying AND ("social media" OR internet OR technology)) OR TX (ostracism AND ("social media" OR internet OR technology)) OR TX (victimi* AND ("social media" OR internet OR technology)) OR TX ("social exclusion" AND ("social media" OR internet OR technology)) OR DE Bullying) AND (TX ("psychiatric disorder*" OR "learning disorder*" OR "learning disab*" OR dyslexia OR acalculia OR adhd OR "attention deficit disorder with hyperactivity" OR anxiety OR "anxiety disorder*" OR "psychiatric comorbidit*" OR suicidality OR "suicidal ideation" OR anorexia OR "anorexia nervosa" OR "conduct disorder*" OR depression OR "depressive disorder*" OR "affective disorder*" OR "major depression" OR "persistent depressive disorder*" OR autism OR asperger OR "Oppositional Defiant Disorder" OR "intellectual disorder" OR "intellectual disability" OR disability OR "inclusive school" OR "special needs" OR "special education" OR neurodevelopment OR cognitive OR tourettes OR dyscalculia OR "borderline personality disorder" OR "autism spectrum disorder" OR "autistic disorder" OR "tourette syndrome" OR "asperger syndrome" OR "cognitive dysfunction" OR "intellectual disability" OR "attention deficit and disruptive behavior disorders") OR DE "Mental Disorders" OR DE "Learning Disabilities" OR DE "Attention Deficit Disorders" OR DE "Anxiety Disorders" OR DE Suicide OR DE "Depression (Psychology)" OR DE "Autism Spectrum Disoders" OR DE "Intellectual Disability" OR DE "Special Education") | 8 |

| *Web of Science* | | |
| --- | --- | --- |
| Search | Search terms | Number of results |
| #1 | TS=(child*) OR TS=(adolescen*) OR TS=(kid*) OR TS=(youth) OR TS=(teenage*) OR TS=("youth mental health") OR TS=("adolescent psychology") OR TS=("child psychology") and Preprint Citation Index  (Exclude – Database) | 7,541,985 |
| #2 | TS=(cyberbully*) OR TS=("online ostraci*") OR TS=("cyber ostraci*") OR TS=("online bully*") OR TS=("cyber victim*") OR TS=("cyber aggress*") OR TS=(cyberperpetrat*) OR TS=(cyberbystander*) OR TS=("cyberbullying bystander*") OR TS=("cyber harassment") OR TS=("cyber abuse") OR TS=("cyber deviance")  OR (AB=(bullying ) AND (AB=("social media") OR AB=(internet) OR AB=(technology)))  OR (AB=(ostracism) AND (AB=("social media") OR AB=(internet) OR AB=(technology)))  OR (AB=(victimi*) AND (AB=("social media") OR AB=(internet) OR AB=(technology)))  OR (AB=("social exclusion") AND (AB=("social media") OR AB=(internet) OR AB=(technology))) and Preprint Citation Index  (Exclude – Database) | 9,362 |
| #3 | TS=(paradigm*) OR TS=("experimental paradigm*") OR TS=("experimental task*") OR TS=(Cyberball) OR TS=(O-Cam) OR TS=(Atimia) OR TS=(ScrollQuest) OR TS=("chat room") OR TS=("ostracism online") OR TS=(recall) OR TS=(No1LikesU!) OR TS=(vignette*) OR TS=("social media vignette*") and Preprint Citation Index  (Exclude – Database) | 822,681 |
| #4 | TS=("psychiatric disorder*") OR TS=("learning disorder*") OR TS=("learning disab*") OR TS=(dyslexia) OR TS=(acalculia) OR TS=(adhd) OR TS=("attention deficit disorder with hyperactivity") OR TS=(anxiety) OR TS=("anxiety disorder*") OR TS=("psychiatric comorbidit*") OR TS=(suicidality) OR TS=("suicidal ideation") OR TS=(anorexia) OR TS=("anorexia nervosa") OR TS=("conduct disorder*") OR TS=(depression) OR TS=("depressive disorder*") OR TS=("affective disorder*") OR TS=("major depression") OR TS=("persistent depressive disorder*") OR TS=(autism) OR TS=(asperger) OR TS=("Oppositional Defiant Disorder") OR TS=("intellectual disorder") OR TS=("intellectual disability") OR TS=(disability) OR TS=("inclusive school") OR TS=("special needs") OR TS=("special education") OR TS=(neurodevelopment) OR TS=(cognitive) OR TS=(tourettes) OR TS=(dyscalculia) OR TS=("borderline personality disorder") OR TS=("autism spectrum disorder") OR TS=("autistic disorder") OR TS=("tourette syndrome") OR TS=("asperger syndrome") OR TS=("cognitive dysfunction") OR TS=("intellectual disability") OR TS=("attention deficit and disruptive behavior disorders") and Preprint Citation Index  (Exclude – Database) | 3,149,174 |
| #1 & #2 & #3 & #4 | (TS=(child*) OR TS=(adolescen*) OR TS=(kid*) OR TS=(youth) OR TS=(teenage*) OR TS=("youth mental health") OR TS=("adolescent psychology") OR TS=("child psychology")) AND (TS=(cyberbully*) OR TS=("online ostraci*") OR TS=("cyber ostraci*") OR TS=("online bully*") OR TS=("cyber victim*") OR TS=("cyber aggress*") OR TS=(cyberperpetrat*) OR TS=(cyberbystander*) OR TS=("cyberbullying bystander*") OR TS=("cyber harassment") OR TS=("cyber abuse") OR TS=("cyber deviance") OR (AB=(bullying ) AND (AB=("social media") OR AB=(internet) OR AB=(technology))) OR (AB=(ostracism) AND (AB=("social media") OR AB=(internet) OR AB=(technology))) OR (AB=(victimi*) AND (AB=("social media") OR AB=(internet) OR AB=(technology))) OR (AB=("social exclusion") AND (AB=("social media") OR AB=(internet) OR AB=(technology)))) AND (TS=(paradigm*) OR TS=("experimental paradigm*") OR TS=("experimental task*") OR TS=(Cyberball) OR TS=(O-Cam) OR TS=(Atimia) OR TS=(ScrollQuest) OR TS=("chat room") OR TS=("ostracism online") OR TS=(recall) OR TS=(No1LikesU!) OR TS=(vignette*) OR TS=("social media vignette*")) AND (TS=("psychiatric disorder*") OR TS=("learning disorder*") OR TS=("learning disab*") OR TS=(dyslexia) OR TS=(acalculia) OR TS=(adhd) OR TS=("attention deficit disorder with hyperactivity") OR TS=(anxiety) OR TS=("anxiety disorder*") OR TS=("psychiatric comorbidit*") OR TS=(suicidality) OR TS=("suicidal ideation") OR TS=(anorexia) OR TS=("anorexia nervosa") OR TS=("conduct disorder*") OR TS=(depression) OR TS=("depressive disorder*") OR TS=("affective disorder*") OR TS=("major depression") OR TS=("persistent depressive disorder*") OR TS=(autism) OR TS=(asperger) OR TS=("Oppositional Defiant Disorder") OR TS=("intellectual disorder") OR TS=("intellectual disability") OR TS=(disability) OR TS=("inclusive school") OR TS=("special needs") OR TS=("special education") OR TS=(neurodevelopment) OR TS=(cognitive) OR TS=(tourettes) OR TS=(dyscalculia) OR TS=("borderline personality disorder") OR TS=("autism spectrum disorder") OR TS=("autistic disorder") OR TS=("tourette syndrome") OR TS=("asperger syndrome") OR TS=("cognitive dysfunction") OR TS=("intellectual disability") OR TS=("attention deficit and disruptive behavior disorders")) | 22 |

| *Cochrane* | | |
| --- | --- | --- |
| Search | Search terms | Number of results |
| #1 | (child* OR adolescen* OR kid* OR youth OR teenage* OR "youth mental health" OR "adolescent psychology" OR "child psychology"):ti,ab,kw | 359,997 |
| #2 | (cyberbully* OR (online NEAR ostraci*) OR (cyber NEAR ostraci*) OR (online NEAR bully*) OR (cyber NEAR victim*) OR (cyber NEAR aggress*) OR cyberperpetrat* OR cyberbystander* OR (cyberbullying NEAR bystander*) OR "cyber harassment" OR "cyber abuse" OR "cyber deviance"):ti,ab,kw OR (bullying AND ("social media" OR internet OR technology)):ti,ab,kw OR (ostracism AND ("social media" OR internet OR technology)):ti,ab,kw OR (victimi* AND ("social media" OR internet OR technology)):ti,ab,kw OR ("social exclusion" AND ("social media" OR internet OR technology)):ti,ab,kw | 190 |
| #3 | (paradigm* OR (experimental NEAR paradigm*) OR (experimental NEAR task*) OR Cyberball OR O-Cam OR Atimia OR ScrollQuest OR "chat room" OR "ostracism online" OR recall OR No1LikesU OR vignette* OR ("social media" NEAR vignette*)):ti,ab,kw | 22,767 |
| #4 | ((psychiatric NEAR disorder*) OR (learning NEAR disorder*) OR (learning NEAR disab*) OR dyslexia OR acalculia OR adhd OR "attention deficit disorder with hyperactivity" OR anxiety OR (anxiety NEAR disorder*) OR (psychiatric NEAR comorbidit*) OR suicidality OR "suicidal ideation" OR anorexia OR "anorexia nervosa" OR (conduct NEAR disorder*) OR depression OR (depressive NEAR disorder*) OR (affective NEAR disorder*) OR "major depression" OR "persistent depressive disorder" OR autism OR asperger OR "Oppositional Defiant Disorder" OR "intellectual disorder" OR "intellectual disability" OR disability OR "inclusive school" OR "special needs" OR "special education" OR neurodevelopment OR cognitive OR tourettes OR dyscalculia OR "borderline personality disorder" OR "autism spectrum disorder" OR "autistic disorder" OR "tourette syndrome" OR "asperger syndrome" OR "cognitive dysfunction" OR "intellectual disability" OR "attention deficit and disruptive behavior disorders"):ti,ab,kw | 255,213 |
| #1 & #2 & #3 & #4 | ((child* OR adolescen* OR kid* OR youth OR teenage* OR "youth mental health" OR "adolescent psychology" OR "child psychology"):ti,ab,kw) AND ((cyberbully* OR (online NEAR ostraci*) OR (cyber NEAR ostraci*) OR (online NEAR bully*) OR (cyber NEAR victim*) OR (cyber NEAR aggress*) OR cyberperpetrat* OR cyberbystander* OR (cyberbullying NEAR bystander*) OR "cyber harassment" OR "cyber abuse" OR "cyber deviance"):ti,ab,kw OR (bullying AND ("social media" OR internet OR technology)):ti,ab,kw OR (ostracism AND ("social media" OR internet OR technology)):ti,ab,kw OR (victimi* AND ("social media" OR internet OR technology)):ti,ab,kw OR ("social exclusion" AND ("social media" OR internet OR technology)):ti,ab,kw) AND ((paradigm* OR (experimental NEAR paradigm*) OR (experimental NEAR task*) OR Cyberball OR O-Cam OR Atimia OR ScrollQuest OR "chat room" OR "ostracism online" OR recall OR No1LikesU OR vignette* OR ("social media" NEAR vignette*)):ti,ab,kw) AND (((psychiatric NEAR disorder*) OR (learning NEAR disorder*) OR (learning NEAR disab*) OR dyslexia OR acalculia OR adhd OR "attention deficit disorder with hyperactivity" OR anxiety OR (anxiety NEAR disorder*) OR (psychiatric NEAR comorbidit*) OR suicidality OR "suicidal ideation" OR anorexia OR "anorexia nervosa" OR (conduct NEAR disorder*) OR depression OR (depressive NEAR disorder*) OR (affective NEAR disorder*) OR "major depression" OR "persistent depressive disorder" OR autism OR asperger OR "Oppositional Defiant Disorder" OR "intellectual disorder" OR "intellectual disability" OR disability OR "inclusive school" OR "special needs" OR "special education" OR neurodevelopment OR cognitive OR tourettes OR dyscalculia OR "borderline personality disorder" OR "autism spectrum disorder" OR "autistic disorder" OR "tourette syndrome" OR "asperger syndrome" OR "cognitive dysfunction" OR "intellectual disability" OR "attention deficit and disruptive behavior disorders"):ti,ab,kw) | 7 |

## Submission Information

Article title: Experimental Investigations of Social Exclusion Among Adolescents with Psychiatric Disorders: A Systematic Review

Journal name: European Child & Adolescent Psychiatry

Author names: Lior Weinreich, Kristina Moll, Matthias F. J. Sperl, Gerd Schulte-Körne, Bert Timmermans

Affiliation and e-mail address of the corresponding author: Department of Child and Adolescent Psychiatry, Psychosomatics and Psychotherapy, University Hospital, Ludwig Maximilian University of Munich, Germany | [Lior.Weinreich@med.uni-muenchen.de](mailto:Lior.Weinreich@med.uni-muenchen.de)

## Filled-out PRISMA checklist

| Section/topic | # | Checklist item | Reported on page # |
| --- | --- | --- | --- |
| **TITLE** |  |  |  |
| Title | 1 | Identify the report as a systematic review, meta-analysis, or both. | 1 |
| **ABSTRACT** |  |  |  |
| Structured summary | 2 | Provide a structured summary including, as applicable: background; objectives; data sources; study eligibility criteria, participants, and interventions; study appraisal and synthesis methods; results; limitations; conclusions and implications of key findings; systematic review registration number. | 2 |
| **INTRODUCTION** |  |  |  |
| Rationale | 3 | Describe the rationale for the review in the context of what is already known. | 3-5 |
| Objectives | 4 | Provide an explicit statement of questions being addressed with reference to participants, interventions, comparisons, outcomes, and study design (PICOS). | 5 |
| **METHODS** |  |  |  |
| Protocol and registration | 5 | Indicate if a review protocol exists, if and where it can be accessed (e.g., Web address), and, if available, provide registration information including registration number. | 6, Appendix |
| Eligibility criteria | 6 | Specify study characteristics (e.g., PICOS, length of follow-up) and report characteristics (e.g., years considered, language, publication status) used as criteria for eligibility, giving rationale. | 5-6 |
| Information sources | 7 | Describe all information sources (e.g., databases with dates of coverage, contact with study authors to identify additional studies) in the search and date last searched. | 6 |
| Search | 8 | Present full electronic search strategy for at least one database, including any limits used, such that it could be repeated. | Appendix |
| Study selection | 9 | State the process for selecting studies (i.e., screening, eligibility, included in systematic review, and, if applicable, included in the meta-analysis). | Appendix |
| Data collection process | 10 | Describe method of data extraction from reports (e.g., piloted forms, independently, in duplicate) and any processes for obtaining and confirming data from investigators. | - |
| Data items | 11 | List and define all variables for which data were sought (e.g., PICOS, funding sources) and any assumptions and simplifications made. | - |
| Risk of bias in individual studies | 12 | Describe methods used for assessing risk of bias of individual studies (including specification of whether this was done at the study or outcome level), and how this information is to be used in any data synthesis. | Appendix |
| Summary measures | 13 | State the principal summary measures (e.g., risk ratio, difference in means). | - |
| Synthesis of results | 14 | Describe the methods of handling data and combining results of studies, if done, including measures of consistency (e.g., I^2^) for each meta-analysis. | - |
| Risk of bias across studies | 15 | Specify any assessment of risk of bias that may affect the cumulative evidence (e.g., publication bias, selective reporting within studies). | - |
| Additional analyses | 16 | Describe methods of additional analyses (e.g., sensitivity or subgroup analyses, meta-regression), if done, indicating which were pre-specified. | - |
| RESULTS |  |  |  |
| Study selection | 17 | Give numbers of studies screened, assessed for eligibility, and included in the review, with reasons for exclusions at each stage, ideally with a flow diagram. | 6-7 |
| Study characteristics | 18 | For each study, present characteristics for which data were extracted (e.g., study size, PICOS, follow-up period) and provide the citations. | 7-8 |
| Risk of bias within studies | 19 | Present data on risk of bias of each study and, if available, any outcome-level assessment (see Item 12). | 8 |
| Results of individual studies | 20 | For all outcomes considered (benefits or harms), present, for each study: (a) simple summary data for each intervention group and (b) effect estimates and confidence intervals, ideally with a forest plot. | 9 |
| Synthesis of results | 21 | Present results of each meta-analysis done, including confidence intervals and measures of consistency. | - |
| Risk of bias across studies | 22 | Present results of any assessment of risk of bias across studies (see Item 15). | - |
| Additional analysis | 23 | Give results of additional analyses, if done (e.g., sensitivity or subgroup analyses, meta-regression) (see Item 16). | - |
| DISCUSSION |  |  |  |
| Summary of evidence | 24 | Summarize the main findings including the strength of evidence for each main outcome; consider their relevance to key groups (e.g., health care providers, users, and policy makers). | 11-14 |
| Limitations | 25 | Discuss limitations at study and outcome level (e.g., risk of bias), and at review level (e.g., incomplete retrieval of identified research, reporting bias). | 14-15 |
| Conclusions | 26 | Provide a general interpretation of the results in the context of other evidence, and implications for future research. | 15 |
| FUNDING |  |  |  |
| Funding | 27 | Describe sources of funding for the systematic review and other support (e.g., supply of data); role of funders for the systematic review. | Statements and Declarations |
